# Impact of Once-Daily Ivabradine on Average Resting Heart Rate in Patients With Reduced Ejection Fraction With Systolic Dysfunction: A Multicenter, Post-marketing Observational Study

**DOI:** 10.7759/cureus.108875

**Published:** 2026-05-15

**Authors:** Ajit Mullasari, Jabir Abdullakutty, Satish Suryavanshi, Milind A Gadkari, Rakesh K Aggrawal, Avanti Sable, Pravin Sawant

**Affiliations:** 1 Cardiology, The Madras Medical Mission, Chennai, IND; 2 Cardiology, Lisie Hospital, Kochi, IND; 3 Cardiology, SMC Heart Institute and IVF Research Centre, Chhattisgarh, IND; 4 Cardiology, King Edward Memorial (KEM) Hospital, Pune, IND; 5 Clinical Research, Deep Heart Center, Ludhiana, IND; 6 Medical Affairs, Abbott Healthcare Pvt. Ltd., Mumbai, IND; 7 Clinical Operations, Abbott Healthcare Pvt. Ltd., Mumbai, IND

**Keywords:** adverse effects, heart failure, heart rate, ivabradine, post-marketing surveillance, systolic, treatment outcome

## Abstract

Background: An elevated resting heart rate (RHR) is associated with an increased risk of morbidity and mortality in patients with cardiovascular (CV) diseases. Twice-daily (BD) dosing of ivabradine effectively lowers RHR and reduces HF-related hospitalizations and mortality. However, benefits depend on dosing frequency, which is vital in ensuring medication adherence.

Aim: This study aims to determine whether a once-daily (OD) prolonged-release formulation of ivabradine maintains effective RHR control over 12 months in patients with HF with reduced ejection fraction (HFrEF) and systolic dysfunction who were previously stabilized on BD ivabradine therapy.

Materials and methods: This was a multicenter, post-marketing, observational clinical study that enrolled 500 patients aged ≥18 years with stable chronic HF, New York Heart Association Class II to III, who were stabilized on a conventional IV ivabradine BD formulation for at least one month. The patients on BD were transitioned to ivabradine OD dosing (10 mg/15 mg) and followed for 12 months (at baseline, 3, 6, 9, and 12 months), with dose adjustments made as required during follow-up. The primary endpoint was the maintenance of a reduced RHR from baseline through 12 months after switching to OD dosing. Secondary endpoints were changes in average RHR at each visit, changes in HR-lowering medication use, and adverse events (AEs). Incidence and time of CV death, all-cause mortality, and hospitalization for worsening HF were prespecified exploratory clinical endpoints.

Results: Of the 500 patients (females: 149, males: 351, mean age: 55.9 ± 11.7 years), 435 patients completed the study. At 12 months, OD ivabradine maintained RHR below baseline, with a mean difference of 3.6 beats per minute (95% confidence interval: -5.058 to -2.338; p < 0.0001). RHR remained consistently below baseline at all follow-up visits, with a significant reduction at each time point (p < 0.0001). Five deaths (1.0%) (four due to CV diseases, one due to a fall) were reported at 12 months. Two patients (0.4%) required hospitalization for worsening HF. The mean time to CV death, all-cause mortality, and hospitalization due to worsening HF were 134.75, 118.8, and 276 days, respectively. AEs were reported in 10 patients (2.0%), and serious AEs in 9 patients (1.8%), all of which were unrelated to study treatment.

Conclusions: Ivabradine administered OD over 12 months effectively maintained RHR in patients previously stabilized on a BD regimen when used alongside other HR-lowering therapies. The observed AE profile was consistent with findings in the published literature, in which most AEs are attributed to underlying conditions or concomitant medications rather than to the drug itself. This supports the overall tolerability and known safety profile of OD dosing of ivabradine, positioning it as a viable and convenient alternative for managing HFrEF.

## Introduction

Heart failure (HF) is a global concern, affecting 56.5 million people worldwide [1]. In India, the incidence of HF is estimated to range between 0.5 and 1.7 cases per 1,000 people annually [2]. The condition is concerning, as it is associated with an increased risk of hospitalization, morbidity, mortality, and significant healthcare costs [3]. According to the Indian College of Cardiology-National Heart Failure Registry [4], HF leads to annual hospitalization in 1% of the general population and 5-10% of the elderly population [4]. Mortality rates are higher in India, accounting for 15-20% compared to 4-5% of the population in the developed countries [4]. HF with reduced ejection fraction (HFrEF), defined as a left ventricular ejection fraction (LVEF) ≤40%, accounts for approximately 50% of all HF cases [5].

HFrEF first-line treatment comprises beta-blockers and angiotensin-converting enzyme inhibitors (ACEis). The MILESTONE registry reports that beta-blockers are the most commonly prescribed drugs by healthcare practitioners (HCPs) to manage resting heart rate (RHR) in HF patients [6]. Despite advancements, HFrEF readmission and mortality rates remain high [7,8]. The European Society of Cardiology (ESC) guidelines recommend managing HF with the target dose or the maximum tolerated dose of ACEIs and beta-blockers; however, many patients in daily practice do not achieve these doses [9,10]. As highlighted in the BIOSTAT-CHF study, receiving <50% of the recommended dose increases the risk of HF-related hospitalization and mortality [11].

Ivabradine is intended to reduce the risk of hospitalization for worsening HF in patients with RHR ≥70 beats per minute (bpm) who are taking maximally tolerated doses of beta-blockers or cannot tolerate them [12]. Ivabradine lowers the heart rate (HR) by selectively inhibiting the funny current (If), which reduces the spontaneous pacemaker activity of the sinoatrial node without affecting ventricular repolarization or myocardial contractility [12]. Thus, HR reduction improves cardiac efficiency in HF primarily by prolonging diastolic filling time. In contrast to beta-blockers, which may reduce LV contractility, ivabradine lowers HR without impairing myocardial contractile function, thereby helping to preserve stable hemodynamics. This mechanism may facilitate favorable cardiac remodeling and improved clinical outcomes [13].

Ivabradine is available in standard doses of 2.5 mg, 5 mg, and 7.5 mg, to be administered orally twice daily (BD). Effective management of HF relies heavily on patient adherence to the prescribed regimen, which is strongly influenced by dosing frequency [13]. Pill burden, discontinuation of medication once positive outcomes are experienced, cost of therapy, and frequency of dosing were the major reasons for nonadherence to the treatment among HFrEF patients [8]. Given this, ivabradine's prolonged-release formulation (10 mg/15 mg) administered once daily (OD) could be a preferable option to the BD regimen, provided it maintains the same effectiveness and safety profile over the 24-hour dosing interval and potentially improves long-term adherence by simplifying to an OD regimen. Although prior studies, such as the PROFICIENT trial, have demonstrated the noninferiority of ivabradine OD compared to ivabradine BD, the study followed patients for only three months, leaving a gap in long-term data [13]. To address this, Abbott approached the Drug Controller General of India to initiate a post-marketing observational study of ivabradine OD to gather long-term data and provide clinicians with robust evidence. In this post-marketing setting, average RHR was selected as the primary outcome because it provides a practical, quantifiable, and continuous assessment of ivabradine’s effect on HR over 12 months, providing insights into long-term effectiveness.

The present study was conducted to evaluate the impact of ivabradine OD on average RHR over 12 months in patients with HFrEF and systolic dysfunction.

## Materials and methods

Study design and ethical considerations

This was a prospective, multicenter, post-marketing observational study conducted between 17 February 2022 and 24 January 2023. The study enrolled patients diagnosed with HFrEF who were receiving concomitant pharmacotherapy and were additionally prescribed an approved prolonged-release OD formulation of ivabradine (10 mg/15 mg) as a part of routine clinical care. A total of 500 patients were enrolled in the study from the outpatient department of 13 study sites across India. The sample size was estimated based on a mean difference of 1.10 and a standard deviation of 8.42, as reported by Mullasari et al. [13]. The effective size was 0.13. Keeping the significance level (α) of 0.05 and a power of 0.80, the analysis initially indicated a sample size of 462 participants. However, considering attrition, the sample size was increased to 500 participants. Ethical approval for the study was obtained from the Institutional Ethics Committee of Vijaya Institute of Clinical and Medical Research before study initiation, with the registration number of ECR/180/Inst/TN/2013/RR-20. The study was conducted in accordance with the Declaration of Helsinki, ICH-E6 R2 Guideline 2016 on “Good Clinical Practice,” New Drugs and Clinical Trials 2019, ICH Guidelines for Good Clinical Practice (1997), and Schedule Y. Written informed consent was also obtained from the patients after explaining the nature and purpose of the study.

Patient selection

Patients with stable chronic HF (New York Heart Association (NYHA) Class II to III) aged 18 years or older, irrespective of gender, and with systolic dysfunction, who were being managed with ivabradine OD as per the approved label, were included in the study. The approved label for ivabradine OD states that patients with HFrEF must be stabilized on a conventional BD formulation of ivabradine (5 mg or 7.5 mg) for at least one month before transitioning to ivabradine OD.

The exclusion criteria comprised patients with hypersensitivity to the active substance or any excipients, acute decompensated HF, pacemaker dependency, RHR below 60 bpm before treatment, severe hepatic impairment, severe hypotension (below 90/50 mmHg), or conditions such as sick sinus syndrome, sinoatrial block, or third-degree atrioventricular block without a functioning demand pacemaker. Additional exclusion criteria were patients currently hospitalized for HF; those with the experience of myocardial infarction or coronary revascularization within the past two months; and patients with a history of or plans for a heart transplant, use of a LV assist device, or dialysis. Other exclusions comprised patients with major recurrent life-threatening cardiac arrhythmias, patients with implantable cardiac devices such as pacemakers or defibrillators, and those diagnosed with atrial fibrillation. Patients with AF or implanted cardiac devices were excluded in line with the approved ivabradine prescribing information, which notes that ivabradine increases the risk of AF and is not recommended in patients with demand pacemakers set to ≥60 bpm, as these patients cannot reliably achieve a target HR <60 bpm. This exclusion ensures a valid assessment of RHR, the primary endpoint, and acknowledges that findings may not be fully generalizable to patients with AF or implanted devices. Furthermore, patients with a history of terminal renal, hepatic, or pulmonary disease, participants involved in other clinical studies with an investigational product, and those with any condition deemed inappropriate for the study by the investigator based on prescribing information were also excluded. Additionally, patients presenting with symptoms of COVID-19 or testing positive for COVID-19 at enrollment or during the study period were excluded, as active infection may acutely affect HR, hemodynamics, and HF outcomes, potentially confounding the assessment of the effect of ivabradine on RHR.

Study procedure and data collection

**Figure 1:**
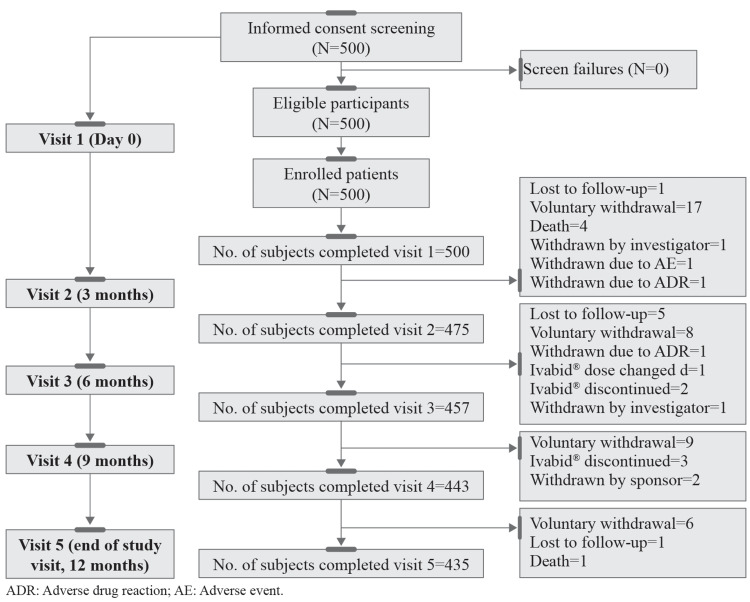
Flow chart representing the disposition of patients in the study AE: adverse event, ADR: adverse drug reaction, No.: number

All patients were followed for 12 months, with screening/enrollment and baseline visits on Day 0 and follow-up visits at 3, 6, 9, and 12 months (window period: ±2 weeks for visits 2-5). The disposition of patients in the study is summarized in Figure 1. Patient adherence to ivabradine OD was not directly measured in this post-marketing observational study. However, RHR, the primary outcome, was monitored at each visit and reflects patients’ treatment exposure over time.

At Baseline

The demographic details of the patients were recorded at baseline (Visit 1). Medical history collected included information on the onset and duration of HF, NYHA class, HF etiology, and any comorbidities. Concomitant medication details comprised both past and current treatments, and if any dose adjustments were required, the reason for the same was noted (if available). Physical examination was conducted, including recording of vital signs (blood pressure, body temperature, respiratory rate, and RHR (both pulse rate and electrocardiogram)) across all sites.

At Follow-up

At Visits 2, 3, 4, and 5 (3, 6, 9, and 12 months, respectively), follow-up information was collected, including a physical examination and details of concomitant medications, including HR-lowering medications (ivabradine and beta-blockers). Any changes, such as the addition or removal of a drug class, switching medications within the same class, or dosage adjustments, were recorded along with the reason for the change, if available.

Outcome assessment

The primary endpoint of the assessment was a change in average RHR from Visit 1 to Visit 5. The secondary endpoint assessment was the change in the average RHR at each visit from baseline, the number of patients changing (addition/deletion of class of drug or change of drug within the same class) in HR-lowering medications during the study period, and the number and proportions of adverse drug reactions (ADRs) and/or other pharmacovigilance-related information about HR-lowering medications. All adverse events (AEs) and serious adverse events (SAEs) were collected as physician-assessed study endpoints to determine their relationship with the study drug. An event was considered to have a reasonable possibility of being related if there was evidence suggesting a causal relationship with the study drug. Conversely, an event was classified as having no reasonable possibility of being related to the drug when there was no evidence to support a causal relationship. The clinical endpoints were incidence and time of cardiovascular (CV) death, all-cause mortality, and hospitalization for worsening HF.

Statistical analysis

Data were analyzed using SAS version 9.4 (SAS Institute, Cary, NC, USA). Descriptive statistics (n, mean, standard deviation, median, minimum, and maximum) were used to summarize continuous variables, and categorical data were presented as counts and percentages with 95% confidence intervals (CIs). Secondary outcomes, including incidence of CV death, all-cause mortality, and hospitalization for worsening HF, were summarized descriptively as the number and proportion of patients experiencing each event. Median times to events reflect observed durations among affected patients; no formal time-to-event analyses were performed. Exposure-adjusted event rates were not calculated because the study was not designed for formal incidence rate estimation, and follow-up duration varied due to treatment discontinuation inherent in real-world clinical practice. The change in outcome parameters from baseline to follow-up visits was analyzed using a repeated-measures analysis of covariance (ANCOVA). Only available data from patients with non-missing observations at each time point were included in the analysis; no imputation was performed for missing values. Patients who discontinued the study were included up to their last available observation. Hospitalizations and deaths were investigator-reported; no independent adjudication was undertaken. The significance was kept at 5%. A reduction of more than 6.5 bpm was considered clinically significant based on the PROFICIENT study [13].

## Results

A total of 500 patients (149 females, 351 males) with a mean age of 55.9 ± 11.7 years were enrolled in the study. A total of 222 patients were on beta-blockers. Of the 500 patients, 65 discontinued the study, while 435 completed it. The reasons for discontinuation are detailed in Figure 1. One patient was discontinued due to noncompliance, four patients were lost to follow-up (did not appear for 2 to 3 subsequent protocol visits without any prior information and were not reachable despite multiple attempts to contact them), 41 patients voluntarily withdrew their consent, and 18 patients were withdrawn due to other reasons that might have affected study outcomes (discontinuation of ivabradine OD or reduction of dose below 10 mg OD). One patient was withdrawn due to SAEs. The demographic details of the patients are summarized in Table 1.

**Table 1:** Demographic details of the patients enrolled in the study Continuous variables are presented as mean ± SD, and categorical variables are presented as numbers (percentages). Weight category as per Asian-Indian cut-off. NYHA: New York Heart Association, SD: standard deviation

Variables	Details
Age (mean ± SD)	
Age	55.9 ± 11.7
Age groups, n (%)	
<40 years	45 (9.0)
40-55 years	191 (38.2)
56-75 years	249 (49.8)
>75 years	15 (3.0)
Gender, n (%)	
Female	149 (29.8%)
Male	351 (70.2%)
Race, n (%)	
Asian	500 (100.0%)
Body measurements	
Weight in kg (mean ± SD)	66.51±10.85
Height in cm (mean ± SD)	159.5±8.59
Weight category, n (%)	
Underweight (≤18.5 kg/m^2^)	9 (1.8)
Normal weight (18.5-22.9 kg/m^2^)	104 (20.8)
Overweight (23-24.9 kg/m^2^)	83 (16.6)
Obesity (≥25 kg/m^2^)	304 (60.8)
NYHA class, n (%)	
NYHA class II	343 (68.6)
NYHA class III	157 (31.4)

Change in average HR

By the end of 12 months, the average RHR decreased from 76.3 ± 12.2 at baseline to 73.0 ± 10.2, showing a significant reduction of 3.6 bpm (95% CI: −5.058 to −2.338; p < 0.0001). The decrease in average RHR from baseline was also observed at each visit (visits 2, 3, and 4). Each visit showed a statistically significant reduction in RHR (p < 0.0001) from baseline Table 2. However, this reduction was not considered clinically significant. A clinically significant reduction in RHR from baseline to visits 2, 3, and 5 was demonstrated in a subgroup of patients with NYHA class III HF. In patients on beta-blockers, the mean RHR decreased by 6.9 bpm from baseline to 12 months (p < 0.001). Mean RHR decreased significantly from baseline to Month 12 in both genders. In males, mean RHR declined from 76.47 ± 12.36 bpm at baseline to 72.85 ± 9.85 bpm at Month 12, while in females it decreased from 75.91 ± 12.02 bpm to 73.38 ± 10.90 bpm. Reductions in RHR at all follow-up visits were statistically significant in both groups (p < 0.0001). Likewise, a significant reduction in mean RHR (1 bpm, p = 0.036) from baseline to 12 months was observed in patients not on beta-blockers (n = 278).

**Table 2:** Change in RHR at baseline and follow-up Continuous variables are presented as mean ± SD and median. The RHR is in bpm. * Significance at p < 0.05. # The mean change in RHR and the significance is calculated based on the number of patients available at each respective time point, compared to their RHR at baseline. bpm: beats per minute, CI: confidence interval, , HR: heart rate, Max: maximum, Min: minimum, RHR: resting heart rate, SD: standard deviation

HR	Visit 1 (baseline)	Visit 2 (3 months)	Visit 3 (6 months)	Visit 4 (9 months)	Visit 5 (12 months)
N	500	475	457	443	435
Mean ± SD	76.3 ± 12.2	71.4 ± 9.7	73.6 ± 10.3	73.5 ± 9.5	73.0 ± 10.2
Median	74.5	69.5	72.8	74	72.5
Min, max	50.5, 115	43, 112	42, 114	45, 112.5	42, 115
Change in RHR from visit 1 (mean ± SD)^#^	-	−5.1 ± 11.8	−3.1 ± 13.9	−3.0 ± 13.2	−3.6 ± 14.4
95% CI for change from baseline	-	-6.057, -3.775	-4.203, -1.562	-4.375, -1.819	-5.058, -2.338
Test value	-	-7.82	-3.47	-4.08	-4.62
Significance (p)	-	<0.0001*	<0.0001*	<0.0001*	<0.0001*

CV death, all-cause mortality, and hospitalization

During the 12-month study period, there were five deaths. Of these, four (0.8%) were attributed to CV diseases, and one (0.2%) was due to a fall. Additionally, two patients (0.4%), with one being admitted twice, required hospitalization for worsening HF. Overall, very few fatalities were reported throughout the study (Table 3). Median time to CV death was 88 days. For all-cause mortality, the median time was 80 days, and the median time for hospitalization due to worsening of HF was 253 days.

**Table 3:** CV death, all-cause mortality, and hospitalization for worsening of HF Categorical variables are presented as numbers (percentages). Percentages are based on “N” for gender and NYHA class. Percentages are based on the total number of patients enrolled (n = 500) for the reasons of the events reported. * The event count for hospitalization due to worsening HF is three but involves only two patients, as one patient was hospitalized on two separate occasions HF: heart failure, LV: left ventricular, N: number of patients, NA: not applicable, NYHA: New York Heart Association

Characteristics	CV death	All-cause mortality	Hospitalization for worsening HF
N	4	5	2*
Reasons, n (%)			
Cardiac arrest	2 (0.4)	2 (0.4)	NA
Cardiogenic shock	1 (0.2)	1 (0.2)	NA
Cardiogenic shock and pulmonary edema, severe LV dysfunction	1 (0.2)	1 (0.2)	NA
Due to fall	NA	1 (0.2)	NA
Hospitalization for worsening HF	NA	NA	3 (0.6)
Gender, n (%)			
Female	0 (0.0)	1 (20.0)	0 (0.0)
Male	4 (100.0)	4 (100.0)	2 (100.0)
NYHA class, n (%)			
NYHA class II	4 (100.0)	4 (100.0)	2 (100.0)
NYHA class III	0 (0.0)	1 (20.0)	0 (0.0)

The mean time to CV death was 134.75 ± 127.69 days. For all-cause mortality, the mean time was 118.80 ± 116.19 days; for hospitalization due to worsening HF, it was 276.00 ± 40.71 days.

Change in HR-lowering medications

Over the course of the 12-month study, most patients did not need their ivabradine dosage changed. Similarly, the proportion of patients requiring ivabradine dose adjustments decreased from 0.4% at baseline to 0.2% at Visit 5, representing a −0.2% change. Patients requiring any HR-lowering medication other than ivabradine dropped from 44.2% at baseline to 42.3% at Visit 5 (−1.9% change). However, there was an increase in patients (0.7%) who required changes to HR-lowering medications other than ivabradine. Discontinuation of a drug class occurred in 0.4% at Visit 3, 0.2% at Visit 4, and 0% at Visit 5. Additionally, only 0.2% of patients required the addition of any drug class, and 0.2% required a change within the same class (Table 4). All changes in HR-lowering medications were investigator-driven.

**Table 4:** Change in HR-lowering medications during the study period Data presented as numbers (percentages). HR: heart rate, n: number of patients

Category	Visit 2 (n = 475)	Visit 3 (n = 457)	Visit 4 (n = 443)	Visit 5 (n = 435)	Absolute percentage-point change from Visit 2 to Visit 5
Patients requiring ivabradine dose change, n (%)	2 (0.4)	1 (0.2)	2 (0.4)	1 (0.2)	−0.2
Patients requiring any HR-lowering medication other than ivabradine, n (%)	210 (44.2)	199 (43.4)	188 (42.2)	184 (42.3)	−1.9
Patients requiring any changes in HR-lowering medication other than ivabradine, n (%)	0 (0.0)	2 (0.4)	2 (0.5)	3 (0.7)	0.7
Patients in whom deletion of any class of drug was done, n (%)	0 (0.0)	2 (0.4)	1 (0.2)	0 (0.0)	0.0
Patients requiring the addition of any class of drug, n (%)	0 (0.0)	1 (0.2)	0 (0.0)	0 (0.0)	0.0
Patients requiring a change of drug within the same class, n (%)	0 (0.0)	1 (0.2)	0 (0.0)	0 (0.0)	0.0

Changes in HR-lowering medication were defined as any dose adjustment, switch of drug within the same class, addition of a new drug class, or deletion/temporary discontinuation of an existing HR-lowering medication. Dose adjustments included any increase or decrease in the prescribed dose of ivabradine or other HR-lowering drugs. Drug switches within the same class involved replacing one medication with another of the same pharmacologic class, while the addition of a new class referred to the initiation of an HR-lowering drug from a different class than previously prescribed. Deletion or temporary discontinuation included stopping a previously prescribed HR-lowering drug either permanently or temporarily.

Adverse events

Only 10 patients experienced 14 AEs (four mild, two moderate, and eight severe) during the study period. All AEs were unrelated to the study treatment. Five events (0.4%) were associated with cardiac disorders, three (0.6%) events with general disorders and administration site conditions, two (0.4%) events with infections and infestations, one (0.2%) event with injury, poisoning, and procedural complications, and three (0.6%) events with respiratory, thoracic, and mediastinal disorders. Furthermore, only two ADRs of mild intensity were reported during the study, both of which resolved without sequelae (Table 5).

**Table 5:** AEs, SAEs, and ADRs reported in the study patients Data presented as numbers (percentages). A patient who might have reported more than one AE has been repeated. ADR: adverse drug reaction, AE: adverse event, COVID-19: coronavirus disease 2019, LV: left ventricular, N: number of patients, SAE: serious adverse event

System organ class	Overall (N = 500)	
	N (%)	Events
AEs		
Number of patients with at least one event	10 (2.8)	14
Cardiac disorders	5 (1.0)	5
Cardiac arrest (led to severe SAEs)	2 (0.4)	2
Cardiogenic shock (led to severe SAEs)	2 (0.4)	2
LV dysfunction (led to severe SAEs)	1 (0.2)	1
General disorders and administration site conditions	3 (0.6)	3
Asthenia	1 (0.2)	1
Death (led to severe SAEs)	1 (0.2)	1
Pyrexia	1 (0.2)	1
Infections and infestations	2 (0.4)	2
COVID-19 (led to moderate SAEs)	2 (0.4)	2
Injury, poisoning, and procedural complications	1 (0.2)	1
Fall (led to severe SAEs)	1 (0.2)	1
Respiratory, thoracic, and mediastinal disorders	3 (0.6)	3
Cough	2 (0.4)	2
Pulmonary edema (led to severe SAEs)	1 (0.2)	1
ADRs		
Number of patients with at least one event	2 (0.4)	2
Dizziness	2 (0.4)	2

Of the 14 AEs, nine led to SAEs (all unrelated to the study treatment). One SAE was of moderate grade, and eight SAEs were of severe grade. Deaths occurred in five patients (two due to cardiac arrest, one due to cardiogenic and pulmonary edema, one due to severe LV dysfunction, and one due to a fall).

## Discussion

Changes in RHR are normal physiological adaptations that help maintain adequate cardiac output; however, excessively elevated HR may be detrimental in patients with underlying HF. A higher HR increases myocardial oxygen demand and shortens diastolic duration, thereby reducing the time available for coronary blood flow and myocardial perfusion. The combination of increased oxygen demand and reduced perfusion time may contribute to myocardial hypoxia and further myocardial injury in patients with HF [14-16]. As a result, lowering and maintaining the lowered RHR is a critical goal in the treatment of HF to improve outcomes. However, many patients with HF also experience low blood pressure, which often coexists with reduced LVEF and further increases the risk of HF-related hospitalizations, CV-related deaths, and all-cause mortality. Managing patients who present with high RHR, low blood pressure, and reduced LVEF simultaneously is particularly challenging for physicians, as it involves balancing complex treatment strategies [16]. The use of ivabradine in the treatment regimen for HF offers significant benefits by effectively lowering RHR without affecting blood pressure [17]. The SHIFT, BEAUTIFUL, and CARVIVA-HF studies have provided evidence of the effectiveness of ivabradine BD in lowering RHR in HF patients [18-20]. These findings provide important context for HR control with ivabradine but do not directly inform the efficacy of OD dosing. Furthermore, adherence to the medical regimen is crucial for optimal HF management, and dosing frequency significantly affects compliance [13]. The current study highlighted both the effectiveness and safety of ivabradine OD in maintaining low RHR in HF patients and is remarkably the first study to determine the long-term impact of ivabradine over 12 months with OD dosing.

In the present study, patients previously stabilized on BD ivabradine received OD dosing, which maintained RHR below baseline over 12 months. The mean RHR decreased by 3.6 bpm at the end of 12 months, with the primary aim of sustaining previously achieved lower HR. In comparison, the PROFICIENT study, a randomized, double-blind, phase 3 trial that transitioned patients from BD dosing to OD dosing of ivabradine, also maintained a lower RHR, with a small reduction of 1.1 bpm from baseline [13]. However, it should be noted that the PROFICIENT trial was short, i.e., only three months, whereas the present study extended over 12 months. Although RHR was maintained and remained statistically lower than baseline, the mean reduction of more than 6.5 bpm is generally considered clinically meaningful [13]. Therefore, while OD dosing effectively sustained the previously achieved RHR, the direct impact of the observed reduction on CV outcomes remains limited.

Furthermore, an apparent increase in RHR between Visits 2 and 3 was observed in the present study, despite continued OD ivabradine therapy. This increase may be due to multiple factors, including normal biological variability, potential changes in treatment adherence or background pharmacotherapy, and evolving clinical status in a real-world population. Importantly, mean RHR remained below baseline values and subsequently stabilized, suggesting sustained effectiveness of ivabradine over time.

Nevertheless, it is important to emphasize that switching to OD dosing of ivabradine is recommended only for patients who have been on the standard BD dosing for at least one month, with a stable condition and an RHR above 50 bpm [13]. Thus, patients receiving OD dosing are already in a stable state, and in such cases, the OD regimen primarily helps maintain the reduced RHR without causing any relapse. The present study demonstrated that the OD regimen successfully supported the long-term management of RHR in stable HF patients without compromising their condition or increasing the risk of relapse. This approach supports ongoing HR management in HF patients while reducing dosing frequency, potentially improving patient compliance. The use of ivabradine OD is also supported by a MILESTONE registry study that reports a greater number of HCPs prescribing it at 3-month follow-ups. At the same time, fewer prefer BD dosing for achieving better treatment adherence and reducing pill burden [6]. This was further supported by a consensus among Indian cardiologists to switch patients from BD to OD dosing once their condition is stabilized [21].

A total of four CV-related deaths, five cases of all-cause mortality, and hospitalization of two patients due to worsening HF with ivabradine OD were reported in the present study. The predominance of events in NYHA class II patients likely reflects the baseline distribution, as more patients were classified as NYHA class II at baseline. In contrast to the present study, the PROFICIENT trial reported no cases of CV-related death, all-cause mortality, or hospitalization for worsening HF at three months [13]. The reduction in mortality and hospitalization rates depends on the baseline risk of developing these outcomes. This was demonstrated in a secondary analysis of the SHIFT trial, which showed that patients with a baseline RHR ≥75 bpm experienced a greater reduction in the primary composite outcomes, including CV death, HF-related death, hospitalization, and all-cause mortality. However, those with a baseline RHR <75 bpm did not show a statistically significant reduction in outcomes, as the drug had less effect on lower-risk conditions [22]. For ivabradine, the modifiable risk increases with higher baseline HR, with the primary composite endpoint progressively increasing by 3% for every 1-bpm rise and by 16% for every 5-bpm rise in baseline HR [23]. The reduced rates of composite outcomes observed over 12 months in the present study demonstrated the effectiveness of ivabradine OD in maintaining CV health, especially after HR stabilization with ivabradine BD. These findings highlighted the importance of a personalized treatment approach, where ongoing management is crucial once initial control has been established.

During the 12-month follow-up, most patients maintained a stable ivabradine dose without adjustments. According to the standard dosing instructions of the United States Food and Drug Administration, for the patients receiving ivabradine for the first time, a 5-mg BD dosing is advised, which is later adjusted by increasing the dose by 2.5 mg BD if RHR is >60 bpm, maintaining the dose if RHR is between 50 bpm and 60 bpm, and decreasing the dose by 2.5 mg BD if RHR is <50 bpm [24,25]. As the present study involved administering ivabradine OD, a prolonged-release formulation of 10 mg or 15 mg was administered. With the baseline RHR already reduced and consistently maintained throughout the study period without clinical variations, no dose adjustments were required. This advantage of OD dosing may be beneficial to patients for treatment adherence and alleviate the need for constant dosage reassessment for HCPs.

The most common AEs associated with ivabradine BD include bradycardia, hypertension, atrial fibrillation, and phosphenes [25]. These AEs were also reported in the SHIFT trial, with 5% of patients having symptomatic bradycardia and 3% having phosphenes [18]. In the present study, about ten patients experienced 14 AEs, of which nine led to SAEs. Mild-intensity ADRs were reported in only two patients. Most of the AEs were cardiac disorders.

Nevertheless, none of the AEs and SAEs were related to the treatment. Furthermore, none of the common AEs reported in the literature and the SHIFT trial were observed in this study. The results of the present study are in accordance with the PROFICIENT trial, which similarly reported minimal AEs with ivabradine OD. Only two SAEs were reported in the PROFICIENT trial, with none being fatal. Furthermore, the trial reported a slightly higher number of events in the BD dosing group compared to the OD dosing group [13]. The lower number of AEs, SAEs, and ADRs in the present study may be partly attributable to the potential link between dosing frequency and lower peak plasma levels, which may lead to fewer AEs with OD administration. According to pharmacokinetic models, multiple doses tend to result in higher plasma drug concentrations compared to a single dose [26]. The reduced plasma concentrations achieved with OD dosing may explain the lower incidence of AEs observed in this study population. However, these pharmacokinetic considerations are hypothesis-generating, as this study did not include pharmacokinetic assessments. Accordingly, the relatively low AEs observed in this study should be interpreted cautiously, as patients' baseline risk profile, prior HR stabilization, optimized background therapy, and study eligibility criteria may also influence them, rather than reflecting an effect of OD ivabradine alone.

While the present analysis focused on clinical outcomes, the social determinants of health, including socioeconomic disadvantage and access to care, are increasingly recognized as important contributors to HF outcomes [27]. Although these factors were not specifically assessed in the present study, their potential influence on real-world HF outcomes warrants further evaluation.

A strength of the present study was its long-term data, extending over 12 months, on the effectiveness and safety of ivabradine OD in a real-world setting, collected through a post-marketing observational study. This extended follow-up provided reliable evidence for HCPs to support the long-term use of ivabradine OD, reinforcing its role in managing patients during the maintenance phase and reducing pill burden. Although this study provided valuable long-term data, it did not include a comparator group. As a post-marketing observational study designed primarily to evaluate the effectiveness and safety of ivabradine OD in routine clinical practice, the absence of a comparator group limits direct comparison with other dosing regimens or therapeutic options and limits causal inference. Clinical outcomes such as CV death, all-cause mortality, and hospitalization were infrequent and analyzed descriptively; therefore, conclusions regarding morbidity or mortality benefits need to be cautiously interpreted. A standardized causality assessment scale was not employed. The study population comprised clinically stable patients who were already on BD ivabradine prior to enrollment, introducing potential selection and survivor bias and limiting extrapolation to patients with advanced HF or ivabradine-naive patients.

Additionally, patients with AF, implanted cardiac devices, and COVID-19 infections were excluded, which may further limit generalizability to the broader HF population. Although concomitant HR-lowering medications were documented, the study did not capture detailed titration patterns or temporal changes in background therapy, which may have influenced RHR and clinical outcomes over time. There was a lack of detailed standardization of RHR measurement across sites, including timing, assessment methods, and resting conditions, which may have introduced inter-site variability. Information on concomitant beta-blocker therapy, whether doses were stable, optimized, or adjusted during follow-up, and its analytical handling were not systematically captured. These factors may have influenced the observed treatment effects. Gender-specific differences in the efficacy of OD ivabradine were not evaluated in this study, limiting insights into potential sex-based variability in treatment response.

Detailed information on concomitant GDMT was not captured. Data on baseline characteristics stratified by study completion status were not systematically collected. Additionally, key clinical variables were not comprehensively captured. Details on covariate adjustment within the repeated-measures ANCOVA model were not systematically captured. Additionally, effect sizes and responder-based outcomes were not reported. While treatment modifications occurred during routine clinical care, the study did not capture structured information on the criteria guiding these changes. Information on patient identification, screening, and transition to OD dosing across centers was not captured, consistent with the study's observational design.

Furthermore, missing-data handling was not formally defined, and data on medication adherence and dose-titration protocols were not recorded. The absence of these details may impact the interpretation of the study outcomes. Direct measures of treatment adherence were not collected; however, RHR assessed at scheduled visits served as an indirect indicator of treatment exposure over time. Approximately 13% of patients discontinued during follow-up. As differences between patients who discontinued and those who completed the study were not formally assessed, the possibility of attrition bias cannot be excluded. Furthermore, as with all post-marketing observational studies, confounding related to patient selection and clinical management cannot be fully excluded.

## Conclusions

In accordance with the approved label, this study initiated ivabradine OD in patients with HFrEF whose HR had been stabilized on the conventional BD formulation. Over a 12-month treatment period, ivabradine OD, administered alongside other HR-lowering medications, effectively maintained RHR below baseline. Both patients on beta-blockers and those not receiving beta-blockers exhibited clinically negligible changes in RHR by Visit 5 (12 months). The minimal incidences of CV deaths, all-cause mortality, and hospitalizations due to worsening HF were consistent with findings from published literature on ivabradine, supporting its known safety and tolerability profile. These results reinforced the potential of ivabradine OD as a convenient and effective alternative to the conventional formulation for managing stable chronic HFrEF. Importantly, this study provided long-term real-world evidence on the effectiveness and safety of ivabradine OD over 12 months. Future real-world studies with an appropriate comparator group and direct assessment of treatment adherence may help further substantiate its effectiveness and safety across different dosing schedules and treatment approaches over the long term.
